# Molecular Modeling of Cardiac Sodium Channel with Mexiletine

**DOI:** 10.3390/membranes12121252

**Published:** 2022-12-10

**Authors:** Boris S. Zhorov

**Affiliations:** 1 Department of Biochemistry and Biomedical Sciences, McMaster University, Hamilton, ON L8S 4K1, Canada; zhorov@mcmaster.ca; 2 Sechenov Institute of Evolutionary Physiology and Biochemistry, Russian Academy of Sciences, 194223 St. Petersburg, Russia; 3 Almazov National Medical Research Centre, 197341 St. Petersburg, Russia

**Keywords:** tonic block, use-dependent block, local anesthetics, molecular modeling, Monte Carlo energy minimizations, voltage-sensing domains, hydrophobic access pathway

## Abstract

A sodium channel blocker mexiletine (MEX) is used to treat chronic pain, myotonia and some arrhythmias. Mutations in the pore domain (PD) of voltage-gated sodium channels differently affect tonic block (TB) and use-dependent block (UDB) by MEX. Previous studies identified several MEX-sensing residues in the hNav1.5 channel and demonstrated that the channel block by MEX increases with activation of the voltage-sensing domain III (VSD_III_), whereas MEX stabilizes the activated state of VSD_III_. Structural rationales for these observations are unclear. Here, Monte Carlo (MC) energy minimizations were used to dock MEX and its more potent analog, Thio-Me2, into the hNav1.5 cryo-EM structure with activated VSDs and presumably inactivated PD. Computations yielded two ensembles of ligand binding poses in close contacts with known MEX-sensing residues in helices S6_III_, S6_IV_ and P1_IV_. In both ensembles, the ligand NH_3_ group approached the cation-attractive site between backbone carbonyls at the outer-pore bottom, while the aromatic ring protruded ether into the inner pore (putative UDB pose) or into the III/IV fenestration (putative TB pose). In silico deactivation of VSD_III_ shifted helices S4–S5_III_, S5_III_, S6_III_ and S6_IV_ and tightened the TB site. In a model with activated VSD_III_ and three resting VSDs, MC-minimized energy profile of MEX pulled from the TB site towards lipids shows a deep local minimum due to interactions with 11 residues in S5_III_, P1_III_, S6_III_ and S6_IV_. The minimum may correspond to an interim binding site for MEX in the hydrophobic path to the TB site along the lipid-exposed sides of repeats III and IV where 15 polar and aromatic residues would attract cationic blockers. The study explains numerous experimental data and suggests the mechanism of allosteric modification of the MEX binding site by VSD_III_.

## 1. Introduction

The inward current through sodium channels Nav1.5 initiates the action potential in cardiomyocytes [1]. Recent cryo-EM structures [2,3,4,5] and the AlphaFold model (Q14524) [6] of the Nav1.5 channel show common structural features of Nav1.x channels many of which have long been proposed in functional studies [7]. The pore-forming α-subunit of the Nav1.x channels folds from a polypeptide chain of four homologous repeat domains (I–IV). Each repeat contains six transmembrane (TM) helices (S1–S6) linked by intra- and extra-cellular loops. A large extracellular membrane-reentering P-loop has membrane-descending helix P1 and membrane-ascending helix P2. In each repeat, helices S1–S4 form a voltage-sensing domain (VSD), whereas helices S5, S6 and the P-loop contribute a quarter to the pore domain (PD). Residues Asp, Glu, Lys, Ala form the selectivity-filter ring DEKA, which divides the ion permeation pathway into the extracellularly exposed outer pore and the inner pore that in open channel joins the cytoplasm. Different conformations of sodium channel are categorized in electrophysiological studies as the resting (closed), open, fast inactivated and slow inactivated states [7].

Interactions of various small-molecule blockers with sodium channels are studied with methods of molecular biology, electrophysiology and molecular modeling [8,9,10,11,12,13,14]. When a channel is activated by infrequent depolarizations of the membrane, a blocker causes so called resting or tonic block (TB). Increasing the stimulation frequency enhances the blocking effect, causing the use-dependent block (UDB) due to the drug access from the cytoplasm through the open activation gate. The maximal channel blockade occurs after a long pre-conditioning depolarization of the membrane that causes a steady-state inactivation of the channel. These phenomena are explained by the modulated receptor hypothesis by Bertil Hille [15] according to which a blocker has the lowest affinity to the closed state, a larger affinity to the open state, and the maximal affinity to the inactivated state. Bertil Hille further proposed that some blockers may reach the pore of the closed channel through the hydrophobic access pathway [15].

A local anesthetics (LA) lidocaine and its elongated derivative, tetracaine, are examples of drugs that inhibit sodium currents by the UDB of the open channels or TB of the resting channels. Tetracaine and lidocaine are predicted to bind in the inner pore of Nav1.x channels and block the ion permeation pathway with the ammonium group approaching the outer pore and the aromatic moiety extending either along the inner pore during UDB or in the III/IV fenestration during TB [16,17]. Crystal and cryo-EM structures of drug-bound sodium channels are consistent with major aspects of these predictions [4,18]. 

A sodium channel blocker mexiletine (MEX) is used to treat chronic pain, myotonia and some arrhythmias. Mutations in sodium channels differently affect UDB and TB by MEX [19]. Inhibition of the hNav1.5 channel by MEX increases with activation of the voltage-sensing domain III (VSD_III_) [20], whereas the bound MEX stabilizes the activated conformation of VSD_III_. Furthermore, for 15 variants of hNav1.5, which are associated with a cardiac arrhythmia (long-QT syndrome), a correlation is found between the voltage dependence of VSD_III_ activation and tonic block of the channel by MEX [20]. Structural rationales for these intriguing observations are unclear.

Here, a cryo-EM structure of the hNav1.5 channel with activated VSDs and partially inactivated PD [4] was used to dock MEX and its more potent analog, Thio-Me2. Hundreds of random starting poses of MEX were generated in the region involving MEX-sensing residues, which are previously identified in mutational studies [19,21]. Monte Carlo energy minimizations yielded ensembles of ligand binding poses that likely represent the TB and UDB block of the channel. In silico deactivation of VSD_III_ tightened the III/IV fenestration, which harbors a part of the TB-bound ligand. The tightening is consistent with analysis of crystal [22] and cryo-EM [2,23] structures of sodium channels with activated and deactivated VSDs.

Among four lipid-exposed sides of the hNav1.5, the side with the III/IV fenestration has the maximal number of polar and aromatic residues that may provide interim binding sites for ligands as they move to the closed channel to achieve the TB block. The MC-minimized energy profile for MEX pulled from the TB site towards the membrane, which was computed in the model with three resting VSDs and activated VSD_III_, shows an energy minimum midway the III/IV fenestration where several channel residues form close contacts with the ligand. Analogous computations for the model with four resting VSDs show the same minimum, but a higher energy barrier between the interim and TB sites. The study explains mutational and structure-activity data and identifies several residues in the III/IV fenestration that may be targets for experimental studies of ligand action on the hNav1.5 channel.

## 2. Methods

The cryo-EM structure of the hNav1.5 channel (PDB ID: 6LQA) obtained at resolution of 3.3 Å [4] was used in this study. Employed methodology of ion channel modeling and ligand docking is described, e.g., in [24,25]. Briefly, the ZMM program (www.zmmsoft.ca) was used to minimize energy in the space of internal (generalized) coordinates with the Monte Carlo (MC) energy minimization method [26] and the AMBER force field [27,28]. Electrostatic interactions were calculated with the distance- and environment-dependent dielectric function [24]. Atomic charges in ligands were calculated with the MOPAC program [29]. Atom-atom interactions were calculated with the distance cutoff of 9 Å and a shifting function [30], but no cutoff was used for electrostatic interactions involving ionized groups.

The MCM sampling protocol randomized the channel side chain torsions as well as positions, orientation and torsion angles of the ligand. During energy minimizations these variables, as well as backbone torsions and bond angles of the ligand were flexible. To prevent large deformations of the channel backbones seen in the cryo-EM structures, “pin” constraints were used. A pin constraint is a flat-bottom parabolic energy function, which imposes an energy penalty when a model C^α^ atom deviates from the template position by >1 Ǻ. The energy penalty was calculated using the force constant of 10 kcal∙mol^−1^∙Å^−2^.

Several protocols were employed for ligand docking. Initially, hundreds random positions and orientations of the ligand were generated with its mass center in the sphere of radius 8 Å located in the pore region accommodating experimentally known MEX-sensing residues. Each stating point was optimized in 100 MC-minimizations without imposing any ligand-channel constraints. A hundred energetically best complexes were further optimized in MCM trajectories that were terminated when 1000 consecutive minimizations did not improve the energy.

For each ligand-channel complex, structures with energy < 7 kcal/mol from the apparent global minimum were clustered in a stack of up to 100 records [24]. Each record in the stack represented a set of complexes with similar positions, orientations, and conformations of the ligand and similar conformations of the channel side chains. (R)-MEX was docked, which in the skeletal muscle fibers produced a stronger UDB and TB of sodium currents than (S)-MEX [31].

In silico deactivating VSD_III_. Methodology of in silico deactivation of VSDs was developed and applied to deactivate VSD_II_ in an insect sodium channel with a scorpion toxin [32], VSD_III_ in a homology model of hNav1.5 [33], and VSD_III_ in a cockroach sodium [34]. Here, the cryo-EM structure of hNa_v_1.5 (PDB ID: 6lqa) with all VSDs activated [4] was used as the starting point for in silico deactivation of VSD_III_. Cryo-EM structures of the rat Na_v_1.5 channel in the apo-form and in complex with the deathstalker scorpion toxin [2] show that the toxin causes a large downshift of helix S4_IV_, significant changes in loop S3–S4_IV_ and in the extracellular half of helix S3_IV_, but rather small shifts of other helices in VSD_IV_. Based on these data, C^α^ atoms in helices S1_III_, S2_III_ and the cytoplasmic half of helix S3_III_, were pinned, while other channel segments were free to move. C^α^ atoms of five basic residues in helix IIIS4 were pulled down through 21 sets of planes (5 planes for five C^α^ atoms in each set), which were normal to the pore axis. Two adjacent planes were 0.5 Å from each other. At each step of the in silico deactivation, the C^α^ atoms of basic residues were free to move within corresponding plane, but not to leave it. The MCM trajectory at each step was terminated when the last 200 consecutive energy minimizations did not improve the apparent global minimum obtained at the step. The starting structure for the next step of S4_III_ downshifting corresponded to the MC-minimized structure found in the previous step. 

Residues in the pore domain are designed by sequential numbers of the hNav1.5 channel (Q14524) and/or by labels, which are universal for P-loop channels [35]. A label contains repeat number, letter “k”, “o”, “p” or “i”, for the linker helix, outer helix, P-loop or inner helix, respectively, and the relative residue number in the segment (Table 1). For example, the outer carboxylate in the first repeat is designated E^1p54^ where the label contains repeat number, character “p” for P-loop, and the residue number relative to position p50 where the selectivity filter residue is located. VSD residues are labeled with their UniProt numbers.

Besides the cryo-EM structures of Nav1.5 channels, crystal structures of the NavAb prokaryotic sodium channel with activated (6p6y) and resting (6p6w) VSDs [22], as well as with the open and closed PD [36] were compared. All the experimental structures were 3D-aligned by minimizing root mean square deviations of C^α^ atoms in P1 helices, the most 3D-conserved parts of P-loop channels, from matching positions in the Kv1.2-Kv2.1 channel (PDB code: 2R9R), the first eukaryotic P-loop potassium channels whose crystal structure was obtained with the resolution below 2.5 Å [38].

Limitations of the employed computational approach should be mentioned. Entropy contributions to the free energy were not computed. Explicit water molecules and lipids were not included. Rather simple treatment of electrostatic interactions was used. Other computational studies with the ZMM program, e.g., [16,39,40] had the same limitations, but their major predictions are confirmed by later published experimental data.

## 3. Results and Discussions

### 3.1. Docking MEX and Its More Potent Analog 

Earlier computational studies with homology models of Nav1.x channels, which are based on crystal structures of prokaryotic P-loop channels, predicted that the ammonium group of LAs approaches the outer pore, while the aromatic ring extends either along the inner pore in the UDB binding pose or along the III/IV fenestration in the TB binding pose [16,17,41]. Cryo-EM structure of the hNav1.5 channel with anti-arrhythmic drug quinidine [4] was used for drug docking. Quinidine and other small-molecule ligands were removed from the cryo-EM structure and coordinates of the apo-channel with presumably inactivated PD and activated VSDs were used for intensive docking of MEX as described in Methods. Hundreds of random starting positions, orientations, and conformations of MEX were generated in the inner-pore region, in the sphere of radius 8 Å with the center at the focus of P1 helices. The region involves MEX-sensing residues L^3i19^, F^4i15^ and S^4p49^ that are identified in mutational studies [19,21]. The docking in this region rather than unbiased docking in the entire channel protein was performed to minimize false-positive binding poses. The later are possible due to the limited precision of force field, ignoring entropy contribution to the free energy, and the lack of explicit water and lipid molecules in the model.

**Figure 1:**
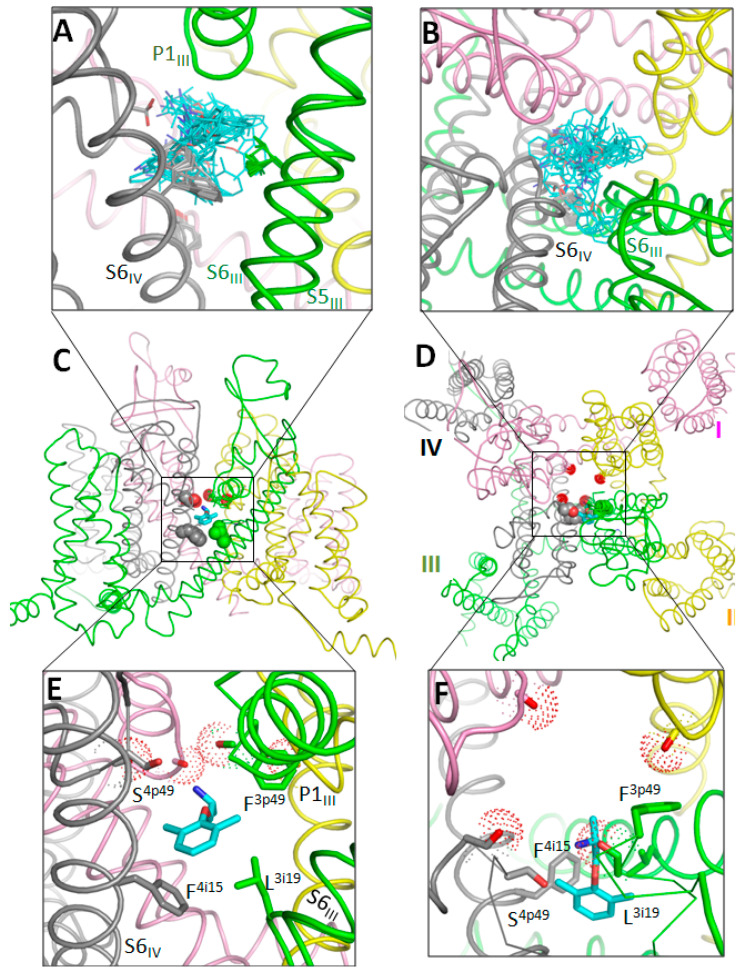
MEX in hNa1.5. Repeats I, II, III and IV are pink, yellow, green and gray, respectively. Carbon atoms in MEX are cyan. (**A**,**B**). Membrane (**A**) and extracellular (**B**) views of top 50 docking poses after refinement of hundreds randomly generates initial structures. (**C**,**D**) Intra-membrane (**C**) and extracellular (**D**) views of the Nav1.5 channel with a representative MEX docking pose in the III/IV fenestration, which presumably corresponds to TB. Space-filled are residues, which are in direct contact with MEX and whose mutations are known to affect the MEX TB. Backbone carbonyls in the outer pore (two positions N-terminal to the DEKA selectivity-filter ring), which create an electronegative site attractive for cationic ligands, are shown by sticks surrounded by dots. (**E**,**F**) Expanded views of panels (**C**,**D**).

Computations yielded a stack with 100 MEX binding poses. Figure 1A,B show the side and extracellular views at the superposition of 50 lowest-energy structures with the ligand-channel interaction energy (enthalpy) within 10 kcal/mol from the apparent global minimum. Structures with the lowest energy include MEX binding poses, which resemble those suggested to represent TB and USB of LAs [16]. Figure 1C–F shows a low-energy structure, which may represent the TB binding pose of MEX. The ligand ammonium group was attracted by the backbone carbonyls of T^3p48^ and T^4p48^, which face the outer-pore bottom. The ligand contacts with backbones are hardly possible to test by mutational analysis. However, the same carbonyls attract the ammonium group of the DEKA lysine K^3p50^ in several cryo-EM strictures of Nav1.x channels, see [42] and references therein. In the cryo-EM structure of hNav1.5, K^3p50^ was in the “dunking” orientation, but during MC-minimizations its ammonium group was repelled by the MEX ammonium group and moved “up” to form a salt bridge with the DEKA glutamate E^2p50^. The aromatic group of MEX partially extended into the III/IV fenestration and formed favorable contacts with L^3i19^ and F^4i15^, whereas the middle part of the ligand interacted with S^4p49^. Mutations of these residues do affect the TB by MEX [19,21]. The TB binding pose of MEX shown in Figure 1E,F resembles that predicted by molecular dynamics simulations of MEX steered through the III/IV fenestration of the NavAb-based model of the hNav1.5 closed channel [43].

**Figure 2:**
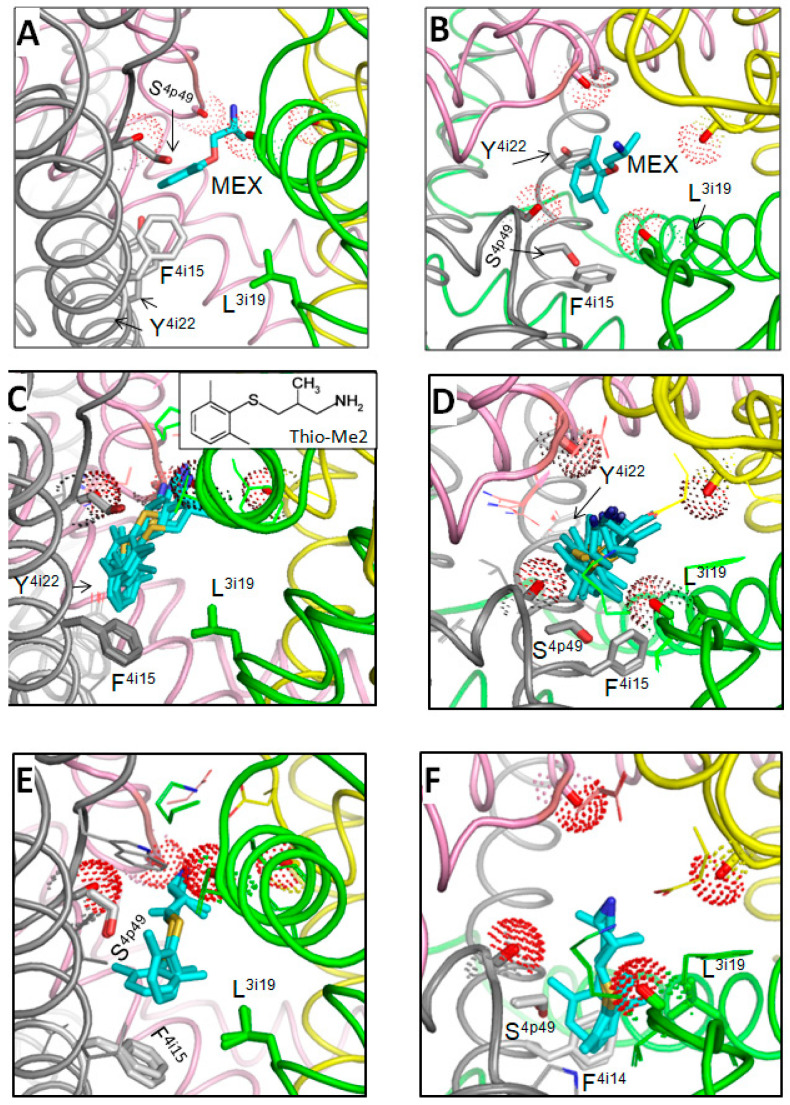
UDB binding pose of MEX and its potent analog, Thio-Me2, in the cryo-EM structure of Nav1.5. Pore-facing backbone carbonyls in position p48 are shown by sticks and dots. (**A**,**B**) Intra-membrane (**A**) and extracellular (**B**), views of the MEX binding pose that corresponds to UDB. The aromatic ring of MEX binds between two aromatic residues in IVS6 in the edge-to-plane mode. The ammonium group deeply penetrates into the outer pore and interacts with the pore-facing backbone carbonyls (red dots) in positions p48 to which the ammonium group of the selectivity-filter lysine is attracted in the ligand-free channel [42]. (**C**,**D**) UDB of Nav1.5 by Thio-Me2. Intra-membrane (**C**) and extracellular (**D**) views of low-energy structures. (**E**,**F**) TB of Nav1.5 by Thio-Me2. Membrane (**E**) and extracellular (**F**) views of low-energy structures.

One of the “vertical” binding poses of MEX, which likely represent the UDB, is shown in Figure 2A,B. The primary ammonium group of MEX deeply penetrated into the outer pore and interacted with backbone carbonyls in positions p48. So deep penetration of the cationic group into the outer pore is hardly possible for the tertiary ammonium group of lidocaine or the quaternary ammonium group of QX-314. This may explain the peculiarities of pharmacological properties of MEX vs. those of lidocaine [44], which are further discussed at the end of this section. The aromatic ring of MEX bound to F^4i15^ and approached Y^4i22^, the residue which is important for UDB by some LAs [8,45]. Mutational data on MEX interaction with Y^4i22^ are unavailable. However, the fact that a MEX derivative with meta-hydroxyl in the aromatic ring is more potent than MEX [46] suggests that the hydroxyl may form an H-bond with Y^4i22^.

Neither TB nor USB binding poses correspond to those proposed in the primarily electrophysiological studies of MEX action on Nav1.5 [19,47]. However, the predicted TB binding pose is consistent with the experimental data [19] according to which mutation F^4i15^A decreased the TB by MEX, mutations N^4i20^A strongly enhanced it, and L^3i19^E somehow enhanced the TB. In the TB binding pose (Figure 1E,F) the aromatic group of MEX is engaged in stacking interactions with F^4i15^ that would be eliminated by mutation F^4i15^A. MEX interactions with L^3i19^ are rather weak, but in the L^3i19^E mutant they would be enhanced by attraction of the aromatic ring edge to the glutamate. Favorable aromatic-carboxylate interactions are revealed by the analysis of crystal structures of many proteins [48]. Mutation N^4i15^A enhanced the closed-channel block (TB), but decreased the open-channel block (UDB) by MEX [19]. This is consistent with the prediction that N^4i15^A would destabilize the open conformation of PD, but stabilize its closed conformation [49,50].

The data that mutation S^4p49^L enhanced the TB by MEX [21] is also consistent with the proposed TB binding pose where engineered S^4p49^L would provide additional hydrophobic contact to MEX (Figure 1E). In the proposed UDB binding pose, MEX interacts with F^4i15^ but it is rather far from L^3i19^ and S^4p49^ (Figure 2A,B). This is consistent with data that mutations F^4i15^A and N^1i20^A decreased UDB, while mutation L^3i19^F enhanced it [19]. The fact that mutation S^4p49^L slightly attenuated the UDB by MEX and accelerated recovery from the block is also consistent with the UDB model where MEX approaches S^4p49^. Mutation S^4p49^L would make the III/IV fenestration more hydrophobic and thus facilitate the drug egress through the fenestration.

A strong UDB and TB are demonstrated for Tio-Me2 [51] that has the elongated sulfur-containing chain between the aromatic ring and the amino group. Docking of Thio-Me2 yielded structures consistent with the UDB (Figure 2C,D) and TB (Figure 2E,F) binding poses of MEX. The longer and more hydrophobic Tio-Me2 would experience stronger interactions with the channel. As compared with the earlier models of Nav1.5-bound lidocaine and tetracaine, which have bulky tertiary amino group [16,17], the protonated primary amino group of MEX and Thio-Me2 penetrated deeper into the outer pore and occupied the site where the primary amino group of the dunked lysine is seen in cryo-EM structures of Nav1.x channel.

The peculiarities of MEX and lidocaine binding pose are consistent with biophysical characteristics of their action on hNav1.5 channels [44]. Thus, (i) both MEX and lidocaine enhanced the slow component of closed-state inactivation, (ii) MEX was a more potent inhibitor than lidocaine, (iii) the recovery from inactivation of Nav1.5 was significantly prolonged by MEX compared to lidocaine, and (iv) MEX displayed a stronger use-dependent inhibition of Nav1.5 than lidocaine. In view of the proposed model, the stronger block by MEX is due to the deeper penetration of the primary amino group into the outer pore as compared to the tertiary amino group in lidocaine. MEX would shift “dunked” DEKA lysine closer to the outer ring of carboxylates thus facilitating closure of the slow-inactivation gate. Due to the same reason, dissociation of the MEX ammonium group from the outer pore would be delayed resulting in delayer recovery from inactivation. 

### 3.2. In Silico Deactivation of VSD_III_

**Figure 3:**
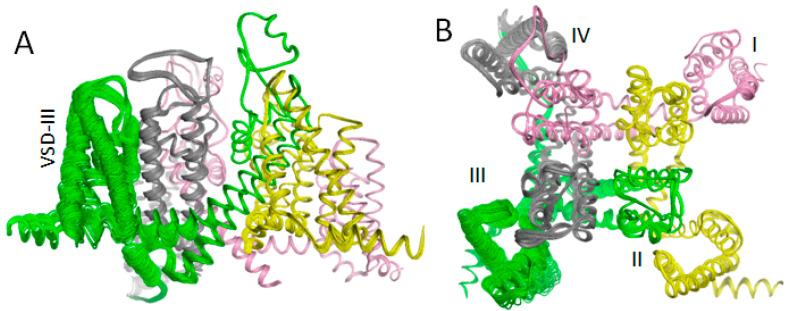
In silico deactivation of VSD_III_. Z-coordinates of C^α^ atoms (z-axis coincides with the pore axis) in S4_III_ arginines were displaced from their positions in hNav1.5 in the cytoplasmic direction with the step of 0.5 Å. At each of the 21 steps the C^α^ atoms were free to move within plane x0y but not to leave it, and the energy was MC-minimized until the last 200 energy minimizations did not improve the apparent global minimum found at the step. No other constraints were imposed. The entire displacements of the z-coordinates of arginines by 10.5 Å were approximately equal to those in the NavAb and NavAb/Nav1.7 experimental structures with up/down positions of VSDs. (**A**,**B**) Intra-membrane (**A**) and extracellular (**B**) views at the superposition of 21 MC-minimized structures. Repeat domains I, II, III and IV are pink, yellow, green, gray, respectively. Note that besides large perturbations in VSD_III_ and IIIS4–S5, significant changes are seen in repeat domain IV, where S5_IV_ directly contacts S4_III_ and S4–S5_III_ wile S6_IV_ contacts S4–S5_III_.

**Figure 4:**
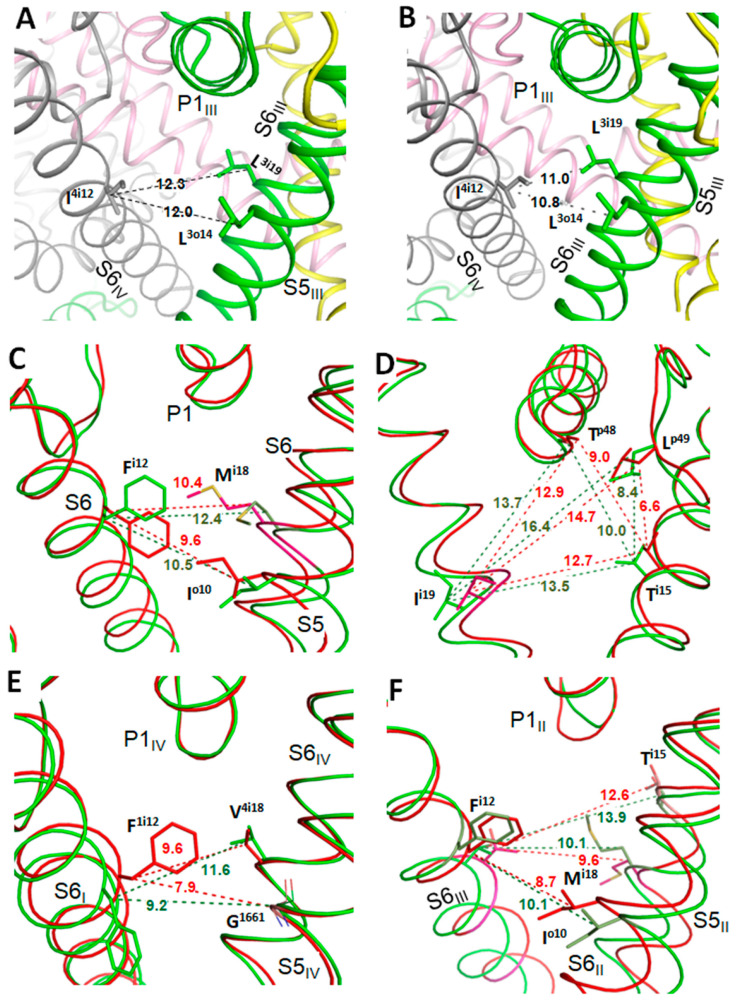
Deactivation of VSDs narrows fenestrations in the pore domain. Channels are viewed from lipids along helix P1 (panels **A**–**C**,**E**,**F**) or from the pore axis (panel **D**). Distances (Å) are shown between C^β^ atoms (C^α^ atom in glycine). In panels (**C**–**F**), structures with the activated and deactivated VSDs are green and red, respectively. (**A**) Cryo-EM structure of hNav1.5. (**B**) hNav1.5 model with in silico deactivated VSD_III_. (**C**) NavAb crystal structure with activated VSDs (PDB ID: 6p6x) and deactivated VSDs (PDB ID: 6p6w). (**D**) Structures from panel *C* viewed from the pore axis. Sticks show side chains of residues, which are in sequentially matching positions with experimentally known MEX-sensing residues in Nav1.5, as well as the backbone carbonyl T^p48^. Activation of VSDs increases the room where MEX bind in eukaryotic Navs. (**E**) Cryo-EM structures of the rNav1.5 channel in the apo form (PDB ID: 6uz3) and with tarantula toxin bound to VSD_IV_ (PDB ID: 7k18). Residues are numbered as in hNav1.5. (**F**) Fenestration II/III in the cry-EM structures of the chimeric NavAb/Nav1.7 channel in the apo state (PDB ID: 6N4Q) and with gating-modifier spider toxin (PDB ID: 6N4R) [23].

The above results support the earlier predicted binding poses of lidocaine and tetracaine that may correspond, respectively, to the UDB and TB in Nav1.5 [16]. The results also explain mutational data on MEX-sensing residues and biophysical characteristics of the channel block by MEX. However, the proposed models do not explain the paradoxical relations between VSD_III_ states and MEX action [20,52]. To address the problem, VSD_III_ was in silico deactivated (Figure 3). The forced downshift of S4_III_ caused significant perturbations in other channel domains, particularly in PD_IV_, due to contacts of helices S4_III_ and S4–S5_III_ with helices S5_IV_ and S6_IV_. Similar perturbations are observed upon in silico deactivation of VSD_III_ in a homology model of hNav1.5 [33] and AlphaFold2 model of the cockroach sodium channel [34]. In silico deactivation of VSD_III_ by downshifting S4_III_ decreased the III/IV fenestration as compared with the cryo-EM structure. The fenestration is lined by several residues, including L^3o14^, L^3i19^ and I^4i12^ (Figure 4A,B). The distances between atom C^β^_I^4i12^ on one hand and atoms C^β^_L^3o14^ and C^β^_L^3i19^ on the other hand in the cryo-EM structure are over 1 Å bigger than in the model with in silico deactivated VSD_III_.

### 3.3. State-Dependent Geometry of Fenestration in Experimental Structures

Similar tendency of widening fenestrations upon activation of VSDs are seen upon comparison of experimental structures of other P-loop channels. Thus, the NavAb channel with VSDs in the resting state is stabilized by engineered disulfide bridges between G^94^C and Q^150^C [22]. Figure 4C,D show that in the crystal structure with VSDs in the activated state (PDB ID: 6p6y), the distances between C^β^ atoms of residues, which line the fenestrations, are up to 2 Å larger than in the crystal structure with VSDs in the resting state (PDB ID: 6p6w). In the rat Nav1.5 channel, VSD_IV_ was trapped in the resting state by the deathstalker scorpion toxin [2]. Figure 4E shows that in the cryo-EM structure of the channel in the apo-state (PDB ID: 6uz3), the distance between C^β^ atoms of residues F^1i12/399^ and V^4i18/1765^ at the opposite sides of fenestration I/IV, is also 2 Å larger than in the structure with the resting VSD_IV_ (PDB ID: 7k18).

VSDs in the homotetrameric chimera channel NavAb/Nav1.7-VS2 with the pore domain from NavAb and VSDs from Nav1.7_VSD_II_ are trapped in the resting state by tarantula toxin m3-Huwentoxin-IV [53]. Distances between C^β^ atoms in diametrically opposed residues of the fenestration decrease upon deactivation of VSDs (Figure 4F). Cryo-EM structures of the NavAb-Nav1.7_VSD_II_ chimera in complexes with the gating modifier toxin ProTx2 bound to the activated and resting VSD_II_ [23] also show significant narrowing of the fenestrations upon VSD deactivation.

Different-width fenestrations in experimental structures of the same channel are likely due to the same mechanisms as seen upon in silico deactivating hNav1.5: contacts of S4–S5 linkers with S5 and S6 helices in the subunit/repeat interface and some downshift of helices S5, which are linked to the S4–S5 helices. The fact that the activated VSD_III_ of Nav1.x channels is stabilized by lidocaine [54,55] or MEX [20], whereas bound ligands stabilize VSD_III_ in the activated state suggests that the states with the activated VSD_III_ and wider III/IV fenestration are more complementary to the ligands than those with deactivated VSD_III_. 

### 3.4. Hydrophobic Access Pathway to the Nav1.5 Inner Pore through III/IV Fenestrations Is Paved by Aromatic and Polar Residues

To achieve the TB block of the resting Nav1.5 channel, LAs are proposed to move through the III/IV fenestration, approach the selectivity filter by the ammonium group and remain in the horizontal position [16]. Such binding pose of ligands in the resting channel is implied [56] and modeled [57] for LAs in the closed NavMs channel and for lidocaine in the NavMs-based model of the Nav1.4 channel [17]. More recently, such a binding pose is shown in the crystal structure of flecainide-bound closed NavAb channel [18] where the ammonium nitrogen of the drug closely approaches the backbone carbonyl in position p48, whereas the trifluoromethyl group approaches T^i15^ on one side of the fenestration and M^i18^ on the opposite side.

Access of cationic drugs such as MEX and lidocaine to the inner pore of the Nav1.x channels through the membrane likely involves deprotonation of the ammonium group and subsequent re-protonation in the cytoplasm or within the pore. However, the permanently charged QX-314 moving through entirely hydrophobic path would encounter a large energy barrier. The fact that mutation S^4p49^L strongly increases TB, attenuates UDB and accelerates recovery from the block by MEX and QX-314 suggests the drugs motion through the III/IV fenestration and lipid-channel interface is accelerated [21]. The TB acceleration by S^4p49^L indicates that the ligand motion through the membrane is not the rate-limiting step for the ligand access, implying that polar or aromatic residues in the lipid-channel interface would provide interim binding sites for the ligand cationic group. The types of lipids that solvate the Nav1.5 channel are unknown, but the channel cryo-EM structure allows visualizing the channel residues, which face the lipids.

**Figure 5:**
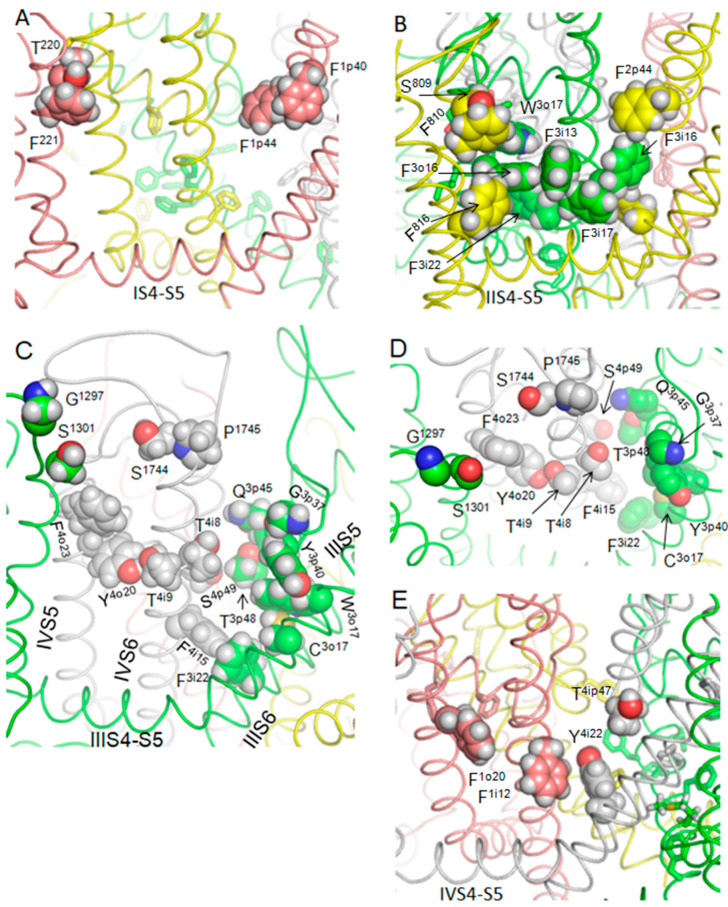
Intra-membrane views at four sides of Nav1.5. (**A**) Interface I/II. (**B**) Interface II/III. (**C**) Interface III/IV. (**D**) Extracellular view of interface III/IV. (**E**) Interface IV/I. Space-filled are lipid-facing polar and aromatic residues, which could attract the ammonium group of MEX in the hydrophobic path from the extracellular space to the pore. Side III/IV has the largest number of polar residues, which may attract polar groups of lipids. MEX could move through the interface between the III/IV side and lipids into the closed-channel pore.

Figure 5 shows lipid-exposed sides of the four fenestrations where polar and aromatic residues could provide interim binding sites for the ligand ammonium group. The lipid-exposed side of fenestration II/III has nine aromatic residues (Figure 5B), whereas the lipid-exposed side of fenestration III/IV has six aromatic residues and seven polar residues (serines, threonines and glutamine). Besides, the backbone oxygens of glycines G^1247^ and G^3p37^, the sidechain nitrogen in P^1745^, and the sulfur atom in C^3o17^ may also attract the permanently charged ammonium group of QX-314 and protonatable amino groups of other ligands. This analysis suggests that the cationic ligands may move from the extracellular space towards the inner pore and III/IV fenestration by hopping through multiple interim binding sites where the ammonium group would be attracted by electronegative atoms and π-electrons in aromatic rings. It should be noted that due to limitations of the MCM methodology, lipid and water molecules were not included in the model. Interactions with lipids may affect side chain conformations of some lipid-exposed residues. However, most of these residues would enjoy interaction with both lipids and hydrophobic moieties of the ligands, whereas aromatic groups and polar atoms in the III/IV fenestration would attract the ligand ammonium group.

### 3.5. State-Dependent Energy Barrier for TB-Bound MEX Egress through Fenestration III/IV

The above analysis illustrates that the III/IV fenestration would provide the access pathway for the ingress and egress of cationic ligands to/from the closed-channel pore. However, it does not explain how activation of VSD_III_ may affect the ligand motion through the fenestration. To address this problem, the cryo-EM structure of hNav1.5 was in silico transformed to yield model VSD_RRRR_ with four VSDs in the resting state and model VSD_RRAR_ with three VSDs in the resting state and VSD_III_ in the activated state. The MEX TB pose (Figure 1) was reproduced in the two models and the ligand was pulled away the pore through the III/IV fenestration by increasing the distance between the pore axis and the para carbon of the ligand aromatic ring. The distance was increased with the step of 0.4 Å and at each step the energy was MC-minimized. 

The channel folding was preserved by pin constraints, which allow C^α^ atoms to deviate penalty-free up to 1 Å from the starting position (see Methods). At each step, the distance between the MEX *para*-carbon and the pore axis was constrained. The constraint did not preclude the *para*-carbon motion over the surface of the sphere whose center was free to translate along the pore axis. In other words, this constraint did not restrict the ligand mobility at the given separation from the pore axis. All side chain torsions and MEX torsion and bond angles were allowed to vary during MC-minimizations. Such setting allowed the flexible MEX molecule to find the energetically optimal egress path, which could be curved. It should be noted that the MEX egress from rather than ingress into the pore was modeled because the starting point for the ingress is undefined. 

**Figure 6:**
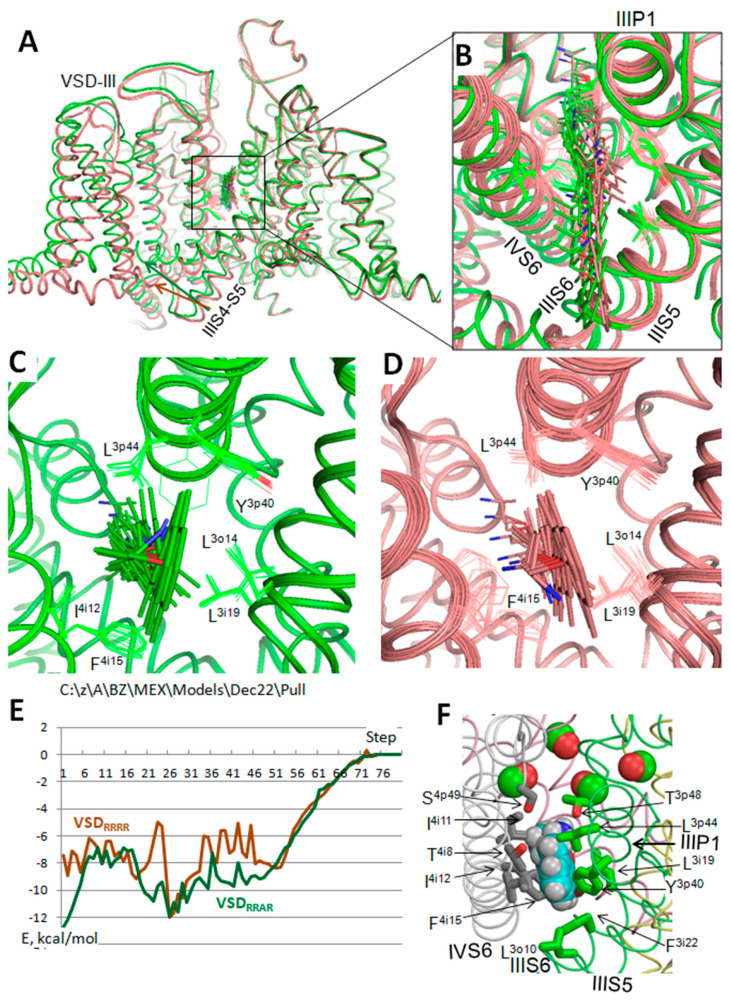
MEX in the III/IV fenestration of hNav1.5. (**A**) Superposition of 80 positions of MEX in model VSD_RRRR_ with four deactivated VSDs (brown) and model VSD_RRAR_ with activated VSD_III_ and three deactivated VSDs (green). The starting position of MEX corresponds to the TB binding pose. Subsequent positions were generated by pulling *p*-carbon of MEX away the pore axis with the step of 0.4 Å. At each step, two MC-minimizations were computed staring from the structure found in the previous step. During the first MCM, backbones were fixed, the *p*–carbon was restrained at the given distance from the pore axis, and all other degrees of freedom were allowed to vary. In the second MCM, no constraints were imposed. Each MCM trajectory was terminated when the last 100 energy minimizations did not decrease the energy of the apparent global minim found at the given step. (**B**) Expanded view of fenestration III/IV along helix P1_III_. Note that in model VSD_RRRR_ fenestration III/IV is narrower than in model VSD_RRAR_. (**C**,**D**) Expanded intra-membrane views of MEX in fenestration III/IV. Side chains of residues whose conformations change with moving MEX are shown by lines. (**E**) Energy of MEX interaction with the channel plotted against the step number. Note that energy of the TB binding pose of MEX in model VSD_RRAR_ is lower than in model VSD_RRRR_ R. (**F**) MEX in model VSD_RRAR_ at step 27, which corresponds to the local energy minimum. Side chains of residues within 4 Å from MEX are shown as sticks. MEX and the pore-facing backbone carbonyls in positions p48 are shown by spheres.

Figure 6A,B show superposed models VSD_RRRR_ (brown) and VSD_RRAR_ (green) with 80 steps of MEX during which the *para*-carbon moved 32 Å away the pore axis from the TB pose. In both models, the egress paths were rather straight but not identical (Figure 6B). The MEX egress in model VSD_RRAR_ did not cause significant deviations of the channel sidechains, including MEX-sensing F^4i15^ and L^3i19^ (Figure 6C). In contrast, the egress of MEX through model VSD_RRRR_ caused significant motion of the F^4i15^ side chain (Figure 6D). The plots of MEX-channel energy (Figure 6E) show that the energy of the starting point (TB pose) in model VSD_RRAR_ is ~5 kcal/mol more preferable than in model VSD_RRRR_, explaining why activation of VSD_III_ stabilizes binding of MEX, whereas TB-bound MEX stabilizes VSD_III_ in the activated state [20]. 

Both models show a minimum of MEX-channel energy at step 27. In model VSD_RRAR_ this is a local energy minimum, which is separated from the global minimum at the TB site by the energy barrier of ~5 kcal/mol. In model VSD_RRRR_ the step 27 corresponds to the global energy minimum along the MEX egress path, which is separated from the TB site by the energy barrier of ~7 kcal/mol. The results suggest that while access of MEX to the closed/inactivated channel with activated VSD_III_ is possible, MEX targeting the closed channel with all VSDs in the resting state is more restricted, but MEX may remain in the interim site at the pathway midpoint until VSD_III_ activates. 

Figure 6F shows MEX in model VSD_RRAR_ midway from the TB binding site to the lipids, at step 27 corresponding to the energy minimum. Residues within 4 Å from the MEX are shown by sticks and backbone carbonyls at the outer-pore bottom are shown by spheres. Besides residues F^4i15^, L^3i19^ and S^4p49^, which are known to affect the TB of MEX, the midway site involves other residues, in particular L^3o10^, L^3i19^, F^3i22^, Y^3p40^, L^3p44^, T^4i8^, I^4i11^ and I^4i12^. Mutational analysis of these residues is beyond the goals of this theoretical study.

### 3.6. Relations between the Half-Voltage of VSD_III_ Activation (^a^V_0.5_) and TB by MEX

In a set of Nav1.5 disease variants, ^a^V_0.5_ of VSD_III_ correlates with TB by MEX [20]: the easier is VSD_III_ activation, the stronger is the TB block. Many of the disease mutations are located in S4_IV_. The fact that these mutations affect the voltage-dependent activation of VSD_III_ seems surprising. The general explanation is suggested by in silico deactivation of VSD_III_, which disturbs VSD_IV_ (Figure 3) due to strong hydrophobic contacts of S4_III_ with S5_IV,_ and contacts of S4–S5_III_ with S5_IV_ and S6_IV_. Similar contacts are described for hNav1. 5 [33] and for an insect sodium channel [34]. On the other hand, contacts with S5_IV_ and S6_IV_ may affect motion of S4–S5_III_ and S4_III_. Structural rationale for the observed decrease in TB by MEX in several disease variants of Nav1.5 [20] are proposed below.

Variant R^1626^P at the extracellular half of S4_IV_ demonstrates the maximal normalized TB of 0.54 and ^a^V_0.5_ of −165 mV. In the cryo-EM structure of hNav1.5, R^1626^ forms a salt bridge with E^1548^ in loop S1–S2_IV_. The salt bridge stabilizes the ”up” state of helix S4_IV_ and thus restricts its motion. Mutation R^1626^P would eliminate the salt bridge and facilitate the motion of S4_IV_ and S4–S5_IV_, thus facilitating activation of S4_III_. The activated VSD_III_ would widen the III/IV fenestration and strengthen TB by MEX.

Variant R^1623^Q, one helical turn above R^1626^, also demonstrates rater strong normalized TB of 0.307. In the cryo-EM structure, R^1623^ forms inter-repeat contact with M^273/1o29^ at the C-end of helix S5_I_. The electrostatic attraction between the guanidinium group of arginine and the sulfur atom of methionine would also stabilize the “up” conformation of S4_III_, but this attraction is weaker than the salt bridge R^1626^----E^1548^.

On the opposite end of the correlation curve TB vs. ^a^V_0.5_ [20], variant M^1652/4o1^R at the N-end of S5_IV_ has the minimal normalized TB by MEX of 0.175 and ^a^V_0.5_ of −102.1 mV. Substitution of M^4o1^R may attract the arginine and S5_IV_ towards the negatively charged inner leaflet of the membrane. Through strong hydrophobic contacts of S5_IV_ with S4_III_ and S4–S5_III_, this motion would shift down the later helices. This would restrain the outward shift of S4_III_, activation of VSD_III_ and keep fenestration III/IV in the narrower state, which disfavors TB. 

VSD_IV_ regulates the onset of fast inactivation, while VSD_III_ determines its recovery [58]. Mutation R^1626^P in S4_IV_ most strongly decelerates inactivation [59]. In the cryo-EM structures of hNav1.5, R^1626^ forms close contacts with M^273^ and N^275^ in loop S5-P1_I_, and it is engaged in electrostatic interactions with D^1550^ in loop S1–S2_IV_. Mutation R^1626^P eliminates these interactions, which stabilize the activated state of S4_IV_ in agreement with the data that activation of S4_IV_ is necessary for fast inactivation [60,61].

### 3.7. Limitations of the Study 

In silico transformation of cryo-EM structures yields models that are less precise than experimental structures. Among multiple low-energy binding poses found for USB and TB by MEX, shown only those where ligands form contacts with experimentally known MEX-sensing residues. Energy profiles of MEX pulled through the III/IV fenestration are just approximate estimates of the enthalpy contribution to the free energy. Nevertheless, the results, which are obtained without biasing contacts of the ligand with specific residues, are consistent with multiple experimental data. 

## 4. Conclusions

Computations suggest that MEX blocks the ion permeation by its ammonium group that competes with sodium ions for the binding site at the outer-pore bottom where four pore-facing backbone carbonyls create a cation-attractive site. The predicted MEX binding modes are consistent with available experimental studies that identified several MEX-sensing residues in hNav1.5. In silico deactivation of VSD_III_ tightens the III/IV fenestration, likely decreasing the TB site affinity to MEX. This may explain the paradoxical data that the channel block by MEX increases with activation of VSD_III_, whereas bound MEX stabilizes the activated state of VSD_III_. Extracellularly applied MEX would reach its binding site in the closed channel by hopping over lipid-exposed polar and aromatic residues in the III/IV domain interface. Along this way, a low-energy interim binding site for MEX is predicted with eleven residues from S5_III_, P1_III_, S6_III_, P1_IV_ and S6_IV_.

## Figures and Tables

**Table 1:** Sequences of transmembrane helices and P-loops in the pore domain of NavAb and hNav1.5 channels ^a^.

Channel	Segment	Residue #	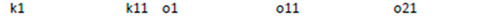
NavAb ^b^	S4-S5, SS	113	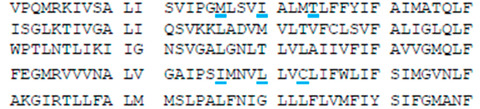
hNa_v_1.5 ^c^	IS4-S5, IS5	232	
	IIS4-S5, IIS5	822	
	IIIS4-S5, IIIS5	1317	
	IVS4-S5, IVS5	1640	
			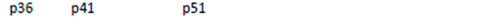
NavAb	P	163	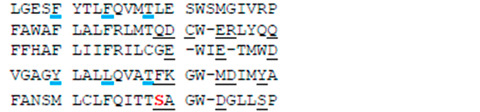
hNa_v_1.5	IP	358	
	IIP	884	
	IIIP	1405	
	IVP	1697	
			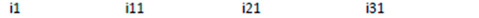
NavAb	S6	192	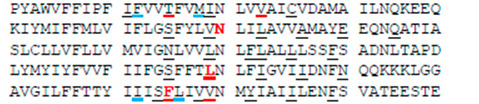
hNa_v_1.5	IS6	387	
	IIS6	913	
	IIIS6	1444	
	IVS6	1746	

^a^ Rows above sequences show residue relative positions in labels, which are universal for P-loop channels [35]. Letters “k”, “o”, “p” and “i” stand, respectively, for the lin¬ker helix (S4–S5), outer helix (S5), P-loop, and ¬inner helix (S6). For example, F^1760^ in helix S6_IV_ of hNav1.5 is designated F^4i15^. ^b^ Voltage-gated sodium channels from *Aliarcobacter butzleri* (A8EVM5_ALIB4). Residues that in the crystal structure of the open channel (PDB ID: 5vb8) [36] face the pore, a fenestration, or both the pore and the fenestration are underlined, respectively, with black, blue or red lines. ^c^ Cardiac voltage-gated sodium channel (SCN5A_HUMAN). Insertions/deletions in the P-loop sequences are according to [37]. Residues whose mutations affect the MEX action [19,21] are red. Residues that in the cryo-EM structure with presumably inactivated pore domain (PDB ID: 6lqa) [4] face the pore, III/IV fenestration or both the pore and III/IV fenestration are underlined, respectively, with black, blue or red lines.

## Abbreviations

LA, local anesthetic; MCM, Monte Carlo minimizations; MEX, Mexiletine; Nav1.5, cardiac voltage-gated sodium channel; PD, Pore domain; TB, Tonic block; UDB, Use-dependent bock; VSD, Voltage-sensing domain.
